# Usefulness of Anticoagulant Therapy in the Prevention of Embolic Complications in Patients with Acute Infective Endocarditis

**DOI:** 10.1155/2014/254187

**Published:** 2014-07-10

**Authors:** Seung-Jae Lee, Sam-Sae Oh, Dal-Soo Lim, Suk-Keun Hong, Rak-Kyeong Choi, Jin-Sik Park

**Affiliations:** ^1^Department of Neurology, Sejong General Hospital, 91-121 Sosabon 2-dong, Sosa-gu, Bucheon-si, Gyeonggi-do 422-711, Republic of Korea; ^2^Department of Thoracic and Cardiovascular Surgery, Sejong General Hospital, Bucheon 422-711, Republic of Korea; ^3^Department of Cardiology, Sejong General Hospital, Bucheon 422-711, Republic of Korea

## Abstract

*Background*. The use of anticoagulant therapy (ACT) in patients with acute infective endocarditis (IE) remains a controversial issue. Our study attempts to estimate the impact of ACT on the occurrence of embolic complications and the usefulness of ACT in the prevention of embolism in IE patients. *Methods*. The present authors analyzed 150 patients with left-sided IE. Embolisms including cerebrovascular events (CVE) and the use of ACT were checked at the time of admission and during hospitalization. *Results*. 57 patients (38.0%) experienced an embolic event. There was no significant difference in the incidence of CVE and in-hospital mortality between patients with and without warfarin use at admission, although warfarin-naïve patients were significantly more likely to have large (>1 cm) and mobile vegetation. In addition, there was no significant difference in the incidence of postadmission embolism and in-hospital death between patients with and without in-hospital ACT. On multivariate logistic regression analysis, ACT at admission was not significantly associated with a lower risk of embolism in patients with IE. *Conclusions*. The role of ACT in the prevention of embolism was limited in IE patients undergoing antibiotic therapy, although it seems to reduce the embolic potential of septic vegetation before treatment.

## 1. Introduction

The use of anticoagulant therapy (ACT) in patients with acute infective endocarditis (IE) remains a controversial issue. Anticoagulation may increase the risk of intracranial hemorrhage (ICH) in IE patients with cerebral septic embolism [1, 2]. In particular,* Staphylococcus* (*S.*)* aureus* IE has been associated with a high risk of septic embolism, ICH, and subsequent mortality [1]. For these reasons, most experts are against the use of ACT in IE patients.

However, discontinuation of warfarin in IE patients with high cardioembolic risk (e.g., prosthetic valve or atrial fibrillation) can increase the probability of intracardiac clot formation and further embolization. A previous study also reported that the incidence of cerebrovascular complications and mortality was increased when ACT was discontinued in patients with prosthetic valve IE [3]. Moreover, some recent studies have shown that warfarin use before IE diagnosis is associated with a reduced risk of cerebral events, suggesting the effectiveness of ACT in the prevention of embolization in IE patients [4, 5].

Based on our clinical experience, the present study attempts to investigate the impact of ACT on the occurrence of embolic complications before and after the initiation of antibiotic treatment and to estimate the usefulness of ACT in the prevention of embolism in IE patients.

## 2. Methods

Using the endocarditis registry of Sejong Cardiovascular Center, the present authors ascertained the names and registry numbers of 299 consecutive patients with suspected IE, who were admitted to the Cardiovascular Center at Sejong General Hospital between May 2000 and May 2013. We then retrospectively reviewed their medical records. From these 299 patients, 108 patients with right-sided IE, 28 with possible left-sided IE, and 13 with incomplete data were excluded. Patients with both-sided IE were classified into the left-sided group. Finally, we analyzed 150 patients who fulfilled the modified Duke's criteria [6] for definite left-sided IE and investigated their detailed clinical information including medical history (age, sex, hypertension, diabetes mellitus, Charlson comorbidity index [7], atrial fibrillation, current smoking status, congestive heart failure (CHF), and history of IE), operation records, computed tomography (CT), magnetic resonance imaging (MRI), echocardiography, and clinical outcomes including mortality. Follow-up data were obtained from outpatient medical records. The study protocol was reviewed and approved by the institutional review board of Sejong General Hospital.

Transthoracic and transesophageal echocardiography were performed in all cases. Echocardiographic data included IE-related valve regurgitation, vegetation length, mobility, and location. Vegetation length was measured in various planes during the first echocardiography and follow-up studies. It was determined whether the maximal vegetation length was >1 cm.

ACT was defined as treatment with warfarin, intravenous unfractionated heparin (UFH), or subcutaneous low-molecular-weight heparin (LMWH). It was checked whether ACT was performed at the time of admission and during the hospitalization. An embolic event was defined as any symptom presumed to be related to septic emboli arising from the vegetation of the infected valve. They included brain complications (cerebrovascular events, mycotic aneurysm, and meningitis), infarct of the spleen or kidney, myocardial ischemia, pulmonary embolism, peripheral artery occlusion, and spondylitis. We inquired about the occurrence of embolic events at the time of admission and during antibiotic therapy.

Cerebrovascular events (CVE) included brain infarcts and ICH. A brain infarct was defined as a focal neurologic deficit of abrupt onset with evidence of new ischemic lesions on brain CT or MRI. ICH was defined as a neurologic symptom with the presence of new intracranial bleeding on CT or MRI and included primary intracerebral hemorrhage (PICH), subarachnoid hemorrhage (SAH), and hemorrhagic infarct (HI).

Statistical analyses were performed with SPSS software, version 18.0 (SPSS Inc., Chicago, IL). The independent* t*-test or Chi-square test (or Fisher exact test) was used to compare the difference between the patient groups with and without ACT. Univariate logistic regression analysis was performed to determine variables related to any embolism during the entire disease period. Multivariate logistic regression was also carried out to assess the influence of warfarin at admission on the embolism. Odds ratio (OR) and 95% confidence interval (CI) were obtained. Kaplan-Meier survival curves were computed according to ACT at admission and during hospitalization. Differences in survival were also estimated using the log-rank test. *P* values <0.05 were considered statistically significant.

## 3. Results

The mean age of 150 patients (101 males and 49 females) included in this study was 48.8 years (range 6–85 years) at admission. There were 139 patients with left-sided IE and 11 with both-sided IE.

*Streptococci*, including viridans species (22 patients, 14.7%), were the most common microorganisms (43 patients, 28.7%), followed by* S. aureus* (20 patients, 13.3%),* Enterococci* (8 patients, 5.3%), and coagulase negative* Staphylococci* (7 patients, 4.7%). In addition, other microorganisms were detected in 37 patients (24.7%), and blood cultures were negative in 35 patients (23.3%).

**Figure 1:**
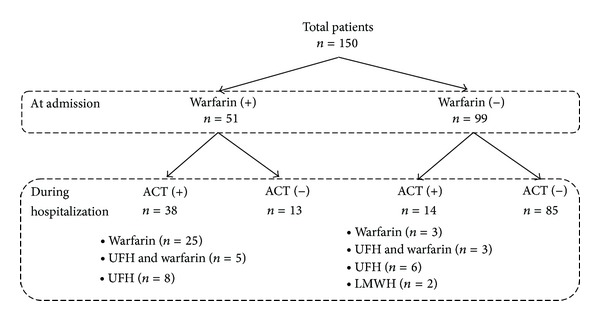
The flowchart of anticoagulant therapy (ACT) at the time of admission and during hospitalization. UFH: unfractionated heparin; LMWH: low-molecular-weight heparin.

Of these 150 patients, 51 patients were on warfarin therapy at the time of admission, while 99 patients were warfarin-naïve. Warfarin use was indicated because of prosthetic heart valves alone (*n* = 20), prosthetic heart valves with atrial fibrillation (*n* = 29), nonvalvular atrial fibrillation (*n* = 1), and atrial fibrillation with mitral regurgitation (*n* = 1). The mean international normalized ratio value (±SD) for the patients on warfarin was 2.91 ± 1.91 at the time of admission, 2.6 ± 0.7 during the first week, and 2.4 ± 0.6 during the second week of hospitalization. Among the 51 patients taking warfarin at admission, 13 patients stopped receiving ACT during the treatment course of IE. ACT was maintained in the other 38 patients during hospitalization (25 on warfarin, 5 on the alternating use of intravenous UFH and warfarin, and 8 on intravenous UFH). In addition, 14 of the 99 patients who were warfarin-naïve at admission received ACT during the treatment period (3 on warfarin, 3 on the alternating use of intravenous UFH and warfarin, 6 on intravenous UFH, and 2 on subcutaneous LMWH) (Figure 1).

Our patients received at least 6 weeks of intravenous antibiotics chosen on the basis of microbial sensitivity, unless a valve operation or death occurred before the end of this period. Of the 150 patients, 35 patients (23.3%) received medical treatment alone, while 115 patients (76.7%) also underwent surgical therapy (53 biologic valves, 39 mechanical valves, 17 valve repairs, and 6 aortic homograft valves).

**Figure 2:**
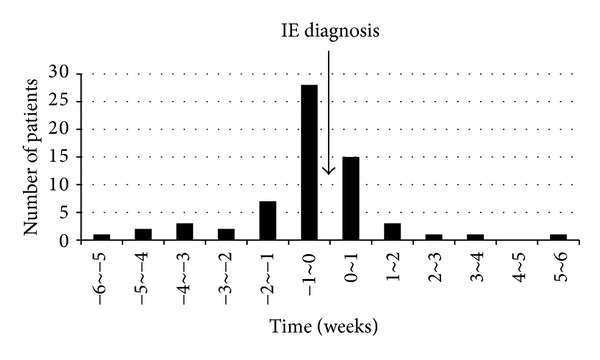
Time distribution of embolic events based on the diagnosis and treatment of infective endocarditis (IE). Negative value indicates the time interval before IE diagnosis.

An embolic event occurred in 57 patients (38.0%). 43 patients (28.7%) had an embolism before antibiotics were initiated and 21 patients (14.0%) had one during antibiotic therapy; embolic events recurred in 7 patients despite antibiotic therapy. Among the 21 patients who had an embolic event during antibiotic therapy, 15 (71.4%) had the event within 1 week after the initiation of antibiotic therapy (Figure 2). A CVE was the most frequent embolic complication related to IE, involving 45 patients (30.0%). Most CVE were brain infarcts, involving 42 patients (28.0%). ICH were found in 15 patients (10.0%), which included 7 cases of PICH, 2 cases of SAH, and 6 cases of HI. In addition, 31 patients had an embolic event in peripheral organs, involving the spleen in 9 patients (6.0%), kidneys in 6 patients (4.0%), heart in 5 patients (3.3%; left anterior descending artery in 4 and right coronary artery in 1), lower limbs in 6 patients (4.0%), spine in 3 patients (2.0%), and lungs in 2 patients (1.3%) with both-sided IE (Table 1).

Table 2 shows the comparison between patients with and without ACT (warfarin) at admission. When compared with the no-ACT group, the ACT group had a significantly higher frequency of cardioembolic risk factors such as atrial fibrillation, history of CHF, and prosthetic valve, whereas the no-ACT group was significantly more likely to have large (>1 cm), mobile vegetation and IE-related valve regurgitation. The period between the initial symptom and diagnosis (diagnostic delay) was shorter in the ACT group, but there was no significant difference in the C-reactive protein level between the two groups. The ACT group showed a statistical trend toward a lower frequency of embolic events at the time of admission and during the entire disease period (*P* < 0.1). However, there was no difference in the incidence of CVE and in-hospital mortality between the two groups.

When the incidence of postadmission embolic complications was compared between patients with and without in-hospital ACT, there was no significant difference in the incidence of embolic events, CVE, or in-hospital death between the two groups. On the other hand, the rate of ICH was higher in the patients with in-hospital ACT, albeit nonsignificantly, than in those without in-hospital ACT (4 out of 52 patients, 7.7%, versus 2 out of 98 patients, 2.0%; *P* = 0.183 by Fisher exact test) (Table 3).

Although ACT was discontinued after admission in 13 out of 51 patients taking warfarin at admission, the incidence of an embolic event did not increase in these patients during the treatment period (1 out of 13, 7.7%, versus 4 out of 38, 10.5%; *P* = 1.000 by Fisher exact test). None of the 13 patients had an ICH during their hospitalization, while 2 out of the remaining 38 patients in whom ACT was continued did. There was, however, no statistically significant difference in the rate of ICH between the two groups (0 out of 13, 0%, versus 2 out of 38, 5.3%; *P* = 1.000 by Fisher exact test).

**Figure 3:**
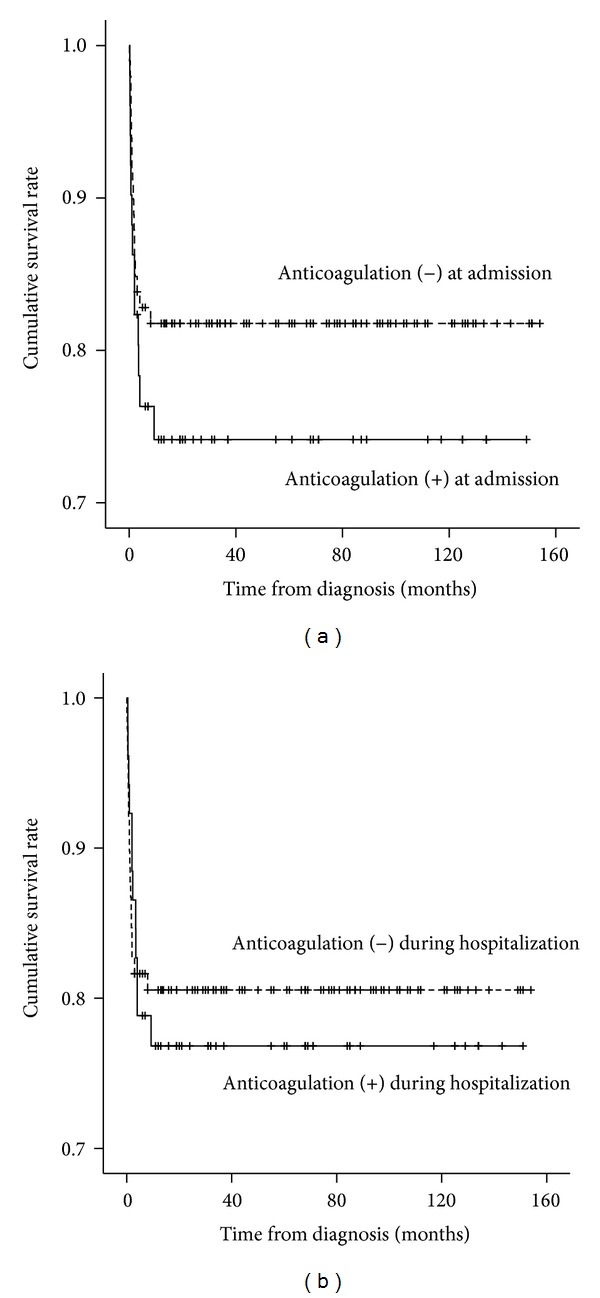
Kaplan-Meier survival curves for patients with and without anticoagulant therapy (ACT). There is no significant difference in survival between patients with and without ACT (*P* > 0.05 by log-rank test).

In addition, there was no significant difference in the long-term survival rate between patients with and without the ACT at admission (*P* = 0.287 by log-rank test) and patients with and without in-hospital ACT (*P* = 0.713 by log-rank test) (Figure 3).

Using logistic regression analysis, we investigated variables related to embolic complications which were developed during the entire disease course. Univariate analysis showed that* S. aureus* infection (*P* = 0.034; OR 2.833; CI 1.080–7.436), large vegetation (>1 cm) (*P* = 0.001; OR 3.066; CI 1.542–6.093), and mobile vegetation (*P* < 0.001; OR 5.584; CI 2.671–11.677) were associated with the occurrence of embolic events. Anticoagulation at admission was associated with a trend toward fewer embolisms, which, however, did not reach statistical significance (*P* = 0.058).

On multivariate logistic regression analysis, atrial fibrillation, prosthetic valve, history of CHF, large vegetation (>1cm), and mobile vegetation were excluded despite their statistical significance in the univariate analysis, because they proved to be closely related to warfarin use at admission, thus having a potential to behave as redundant variables (multicollinearity). The multivariate analysis (using the variables of age, mitral valve IE,* S. aureus* infection, and ACT at admission) demonstrated that* S. aureus* infection independently predicted an embolic event (*P* = 0.033; OR 2.947; CI 1.090–7.965), but ACT at admission was associated with only a trend toward a lower risk of embolism that did not reach statistical significance (*P* = 0.065; OR 0.488; CI 0.228–1.047) (Table 4).

## 4. Discussion

Embolization is a frequent complication which is clinically apparent in up to 50% of IE patients and is known to be associated with* S. aureus* infection, large (>1 cm) vegetation, and mitral valve IE [8–10]. The most common site of embolism in left-sided IE is the brain [10]. Although brain embolisms mostly cause infarcts, it also can cause ICH. In this study, a CVE occurred in 45 of 150 total patients (30%). Of these, 15 patients (10%) had an ICH. A mycotic aneurysm was confirmed in one case. The rates of total CVE and ICH in our study were similar to those reported in previous studies [8–11].

It is important to take measures for the prevention of brain embolism, which may predict poor prognosis in IE patients [8, 11, 12]. Prompt initiation of antibiotic therapy has been the most effective strategy to reduce the rate of septic embolism [13, 14]. Our study also showed that the rates of total embolic events and CVE were decreased to half of their initial incidence after initiation of antibiotics (from 28.7 to 14% and from 20 to 10%, resp.).

However, the ACT as a strategy to prevent embolic events has been the focus of hot debate and discussion in treating IE patients, especially those already on anticoagulation [15–17] due to the reason of increased probability of ICH. This is considered to be due to the fact that a large infarcted area is liable to be transformed into a hemorrhage, and septic emboli lodging inside the vascular lumen can lead to acute erosive arteritis with subsequent arterial rupture. Rarely, a mycotic aneurysm can rupture after its subacute development due to the weakening of the infected vessel wall [18]. Some previous reports also showed an increased risk of cerebral hemorrhage and mortality in IE patients on anticoagulation [1, 2]. In particular, brain complications including fatal ICH are known to be frequent in those infected with* S. aureus*, which is the main reason to avoid anticoagulation in IE patients [2, 8, 13, 17].

On the contrary, more recent studies have suggested a reduced risk of brain embolism without significant hemorrhagic complications in patients on anticoagulation [4, 5]. The authors of these studies argue that the risk of ICH related to anticoagulation has been overestimated and ACT is not associated with ICH, instead having a protective effect against embolic events in IE patients. Theoretically, anticoagulants or antiplatelets seem to suppress the growth of septic vegetation in that its formation is based on fibrin and platelet deposition where bacteria can proliferate and evade the host immune system [19]. In line with the results of their studies, ACT at admission was significantly related to a reduced embolic potential (size and mobility) of vegetation and tended to reduce the rate of embolism related to IE in our patients.

However, Our results showed that ICH was not a rare complication of IE, accounting for 10% of our patients. Additionally, the ACT during antibiotic therapy had no significant association with a lower rate of postadmission embolism, instead showing relatively higher rate of ICH (albeit nonsignificant) during hospitalization in this study. Multivariate regression analysis of our study also demonstrated that ACT at admission showed only a trend toward less risk of IE-related embolism. These results may suggest that “bacterial factors” may have a higher contribution to the formation and growth of “septic” vegetation than “coagulation factors” do, and the effect of ACT on “septic” vegetation thus seems to become more limited after initiation of antibiotic therapy. This presumption can be supported by our results indicating that the occurrence of postadmission embolism did not increase in patients already on warfarin, although ACT was discontinued in those patients after admission. Furthermore, a previous report also concluded that the protective effect of ACT against embolization is likely to disappear after the initiation of antibiotics [4].

Consequently, the overall appropriateness of ACT use should be estimated during IE treatment. Its use should be withheld in those at high risk of hemorrhagic complications. The recently updated guidelines of the American College of Chest Physicians also recommend that ACT not be routinely indicated in IE and warfarin be discontinued at the time of the disease presentation until it is clear that the patient has stabilized without the signs of CNS involvement [20].

Particularly in IE patients with large brain infarcts, ACT should be stopped because it can put these patients at risk of catastrophic ICH [21]. In addition, patients who are found to have microbleeds on T2∗ MRI should be anticoagulated more cautiously because these lesions may signify the presence of unruptured mycotic aneurysms [22] and may also independently predict the subsequent development of symptomatic ICH [23].

Moreover, ACT is not usually recommended for acute treatment of ischemic stroke because evidence to date has demonstrated that ACT, which may increase the risk of bleeding, does not significantly lessen the risk of early neurological worsening or recurrent stroke, even in the cardioembolic stroke subtype [24, 25].

There were several limitations in our study. First, it was based on the experience of a single referral cardiovascular center. The rates of valve operation and culture-negative IE (presumably due to antibiotic use before referral) were higher compared with the results of other studies [4, 8, 9, 11, 12]. Therefore, selection bias is likely to have been present. Second, as an observational retrospective study, patient management (imaging study or the use of ACT) could not be controlled according to a standardized protocol. Thus, an individualized decision making for patient treatment could have an effect on the results of this study. Third, the sample size was limited, although our data of patients had been collected for 13 years. In particular, some subgroup analysis, for example, comparison of patients with and without the continuance of anticoagulants after admission, may not be statistically reliable owing to the small number of patients.

## 5. Conclusions

The role of ACT in the prevention of embolism was limited in IE patients undergoing antibiotic therapy, although it seems to reduce the embolic potential of septic vegetation before antibiotic treatment is started. Instead, ACT in patients on antibiotic treatment for IE may increase the risk of hemorrhagic complications. Therefore, once antibiotic therapy is initiated, the usefulness of ACT should be weighed against its potential risk of devastating ICH, particularly in patients already on ACT.

## Figures and Tables

**Table 1:** Embolic complications of IE patients.

	At admission *N* = 150	During antibiotic therapy *N* = 150	Total
Any embolism	43 (28.7)	21 (14.0)	57 (38.0)
Cerebrovascular events	30 (20.0)	15 (10.0)	45 (30.0)
Brain infarct	28 (18.7)	14 (9.3)	42 (28.0)
ICH	9 (6.0)	6 (4.0)	15 (10.0)
PICH	4 (2.7)	3 (2.0)	7 (4.7)
SAH	1 (0.7)	1 (0.7)	2 (1.3)
HI	4 (2.7)	2 (1.3)	6 (4.0)
Mycotic aneurysm	—	1 (0.7)	1 (0.7)
Meningitis	1 (0.7)	—	1 (0.7)
Splenic infarct	7 (4.7)	2 (1.3)	9 (6.0)
Renal infarct	5 (3.3)	1 (0.7)	6 (4.0)
MI	3 (2.0)	2 (1.3)	5 (3.3)
Pulmonary embolism	1 (0.7)	2 (1.3)	2 (1.3)
Peripheral artery embolism	4 (2.7)	3 (2.0)	6 (4.0)
Spondylitis	3 (2.0)	—	3 (2.0)

ICH: intracranial hemorrhage; PICH: primary intracerebral hemorrhage; SAH: subarachnoid hemorrhage; HI: hemorrhagic infarct; MI: myocardial infarction.

**Table 2:** Characteristics of patients with and without ACT at admission: *n* (%).

	ACT (−) *N* = 99	ACT (+) *N* = 51	*P* value
Age ≥ 65	15 (15.2)	14 (27.5)	0.071
Male	68 (68.7)	33 (64.7)	0.622
Hypertension	20 (20.2)	6 (11.8)	0.196
Diabetes mellitus	18 (18.2)	10 (19.6)	0.832
Atrial fibrillation	12 (12.1)	31 (60.8)	<0.001
Smoking	14 (14.1)	7 (13.7)	0.945
History of IE	1 (1.0)	1 (2.0)	1.000
Dialysis	0 (0.0)	2 (3.9)	0.114
History of CHF	15 (15.2)	28 (54.9)	<0.001
Comorbidity index > 2	17 (17.2)	15 (29.4)	0.083
Prosthetic valve	15 (15.2)	49 (96.1)	<0.001
Mitral valve IE	77 (77.8)	36 (70.6)	0.333
Aortic valve IE	46 (46.5)	26 (51.0)	0.600
Dual valve IE	24 (24.2)	10 (19.6)	0.521
*Staphylococcus aureus *	12 (12.1)	8 (15.7)	0.543
CRP mg/dL	7.2 ± 6.2	8.7 ± 8.3	0.393
Diagnostic delay, days	38.7 ± 38.8	18.6 ± 18.9	<0.001
Echocardiographic findings			
Vegetation > 1 cm	58 (58.6)	14 (27.5)	<0.001
Mobile vegetation	56 (56.6)	20 (39.2)	0.044
Paravalvular infection	28 (28.3)	12 (23.5)	0.533
IE-related valve regurgitation	91 (91.9)	30 (58.8)	<0.001
At admission			
Any embolism	33 (33.3)	10 (19.6)	0.078
Any cerebrovascular event	21 (21.2)	9 (17.6)	0.605
Brain infarct	20 (20.2)	8 (15.7)	0.501
Intracranial hemorrhage	4 (4.0)	5 (9.8)	0.274
During the entire disease period			
Any embolism	43 (43.4)	14 (27.5)	0.056
Any cerebrovascular event	30 (30.3)	15 (29.4)	0.910
In-hospital mortality	16 (16.2)	13 (25.5)	0.171

ACT: anticoagulant therapy; IE: infective endocarditis; CHF: congestive heart failure; CRP: C-reactive protein.

**Table 3:** Comparison of postadmission embolism between patients with and without in-hospital ACT.

	ACT (−) *N* = 98	ACT (+) *N* = 52	*P* value
Any embolism	14 (14.3)	7 (13.5)	0.890
Any cerebrovascular events	8 (8.2)	7 (13.5)	0.303
Brain infarct	7 (7.1)	7 (13.5)	0.205
ICH	2 (2.0)	4 (7.7)	0.183
In-hospital mortality	18 (18.4)	11 (21.2)	0.681

ACT: anticoagulant therapy; ICH: intracranial hemorrhage.

**Table 4:** Factors associated with embolism in patients with IE.

	Univariate analysis	*P *	Multivariate analysis	*P *
	OR (95% CI)		OR (95% CI)	
Age ≥ 65 years	0.560 (0.230–1.365)	0.202	0.657 (0.260–1.665)	0.376
Male gender	1.409 (0.688–2.887)	0.348		
Hypertension	1.024 (0.429–2.443)	0.957		
Diabetes mellitus	0.730 (0.305–1.747)	0.480		
Atrial fibrillation	0.719 (0.341–1.515)	0.385		
History of CHF	0.620 (0.291–1.322)	0.216		
Comorbidity index > 2	0.973 (0.435–2.180)	0.948		
Prosthetic valves	0.472 (0.236–0.941)	0.033		
Mitral valve IE	1.623 (0.730–3.607)	0.235	1.397 (0.612–3.187)	0.427
Aortic valve IE	0.765 (0.394–1.483)	0.427		
Vegetation > 1 cm	3.066 (1.542–6.093)	0.001		
Mobile vegetation	5.584 (2.671–11.677)	<0.001		
*Staphylococcus aureus *	2.833 (1.080–7.436)	0.034	2.947 (1.090–7.965)	0.033
Warfarin at admission	0.493 (0.237–1.025)	0.058	0.488 (0.228–1.047)	0.065

CHF: congestive heart failure; IE: infective endocarditis.
